# Blood-Brain Glucose Transfer in Alzheimer’s disease: Effect of GLP-1 Analog Treatment

**DOI:** 10.1038/s41598-017-17718-y

**Published:** 2017-12-13

**Authors:** Michael Gejl, Birgitte Brock, Lærke Egefjord, Kim Vang, Jørgen Rungby, Albert Gjedde

**Affiliations:** 1 Institute of Biomedicine, Aarhus University, Aarhus, Denmark; 2 Department of Nuclear Medicine and PET Center, Aarhus University Hospital, Aarhus, Denmark; 3 Department of Clinical Biochemistry, Aarhus University Hospital, Aarhus, Denmark; 4 Departments of Endocrinology, Bispebjerg University Hospital, Copenhagen, Denmark and Aarhus University Hospital, Aarhus, Denmark; 5 Department of Neuroscience and Pharmacology, University of Copenhagen, Copenhagen, Denmark; 6 Department of Nuclear Medicine, Odense University Hospital, Odense, Denmark

## Abstract

There are fewer than normal glucose transporters at the blood-brain barrier (BBB) in Alzheimer’s disease (AD). When reduced expression of transporters aggravates the symptoms of AD, the transporters become a potential target of therapy. The incretin hormone GLP-1 prevents the decline of cerebral metabolic rate for glucose (CMR_glc_) in AD, and GLP-1 may serve to raise transporter numbers. We hypothesized that the GLP-1 analog liraglutide would prevent the decline of CMR_glc_ in AD by raising blood-brain glucose transfer, depending on the duration of disease. We randomized 38 patients with AD to treatment with liraglutide (n = 18) or placebo (n = 20) for 6 months, and determined the blood-brain glucose transfer capacity (*T*_max_) in the two groups and a healthy age matched control group (n = 6). In both AD groups at baseline, *T*_max_ estimates correlated inversely with the duration of AD, as did the estimates of CMR_glc_ that in turn were positively correlated with cognition. The GLP-1 analog treatment, compared to placebo, highly significantly raised the *T*_max_ estimates of cerebral cortex from 0.72 to 1.1 umol/g/min, equal to *T*_max_ estimates in healthy volunteers. The result is consistent with the claim that GLP-1 analog treatment restores glucose transport at the BBB.

## Introduction

Aging populations suffer from increased incidence of metabolic disorders and type 2 diabetes, including vascular disease and dementia^1^. Under normal circumstances, glucose molecules in the circulation enter brain tissue from plasma by facilitated diffusion across the two membranes of the endothelium of the blood–brain barrier (BBB). The facilitated diffusion is mediated mainly by the glucose transporter 1 (GLUT1), a key regulator of glucose transport into and out of the brain where it serves to maintain homeostasis^2^. Among the insulin-insensitive transporters, GLUT1 is abundant in the BBB and in astrocytes, while neurons primarily are served by the glucose transporter GLUT3. Brain glucose uptake correlates with GLUT1 levels in cerebral microvessels^3–5^, although glucose transporters other than GLUT1 are known also to operate in the BBB, including in particular the insulin-sensitive glucose transporter GLUT4^6^. Although GLUT1 transport mechanistically is insulin insensitive, GLUT1 transport in general may be said to moderately sensitive to the presence of insulin in the sense that the presence of insulin in earlier studies led to a limited increase in the number of GLUT1 transporters in cell membranes in specific parts of the body (muscle, thyroid gland^7,8^).

Alzheimer’s disease (AD) is characterized by deposits of amyloid-β (Aβ) and hyperphosphorylated tau proteins that may impair neurovascular regulation, blood brain barrier (BBB) integrity^9^, and expression of glucose transporters at the BBB^6^. The deposits also may aggravate the cerebral metabolic decline^10^ that correlates with impaired cognition^11^. Loss of glucose transporters from the BBB is known to occur before the onset of AD symptoms^6^, and PET of [^18^F]fluoro-2-deoxyglucose ([^18^F]FDG)-derived radioactivity in brain reveals progressive reduction of cerebral metabolic rate of glucose CMR_glc_, before detection of clinical symptoms in individuals with subsequently confirmed AD^12^. The diminished metabolism exceeds and precedes brain atrophy and neurodegeneration^13–16^.

There is evidence in AD that impaired brain glucose uptake or metabolism, or both, may be the result of brain insulin resistance, or down-regulation of the glucose transporters in brain required for cerebral glucose uptake and metabolism^17–19^. This means that it is possible that glucose transport across the BBB may become the rate-limiting step of glucose metabolism in AD^20^, the disorder in which the number of glucose transporters is known to be reduced^21,22^. Thus, the decline of the CMR_glc_ may follow the reduction of BBB glucose transport capacity^23,24^. Recent work in mice reveals that reduced expression of GLUT1 in the BBB leads to massive progression of AD neuropathology, and that GLUT1 deficiency in the endothelium initiates BBB breakdown, possibly as an early pathogenic step in the evolution of AD^25^.

The formal differences between the maximum blood-brain glucose transport capacity (*T*_max_) and CMR_glc_ steps of the pathways of glucose metabolism mean that the two measures are unrelated in principle, as the *T*_max_ step depends on the density and activity of transporters, while the CMR_glc_ step depends on and is close to the maximum velocity (*V*_max_) of hexokinase in brain tissue. The phosphorylation of glucose (or [^18^F]FDG) requires the presence of the substrate in the tissue at a concentration consistent with the *V*_max_ of hexokinase, but there is no other link between the two measures. The absence of additional links was the motivation for the analysis presented here, i.e., the determination of the transport changes potentially correlated with the progression of AD.

Recent findings by the present group of researchers show that native GLP-1 raises maximum glucose transport capacity in brain capillary endothelium in healthy humans^2,26^. In the present investigation, we hypothesized that the same mechanism activated by the GLP-1 analog liraglutide may act to prevent the decline of glucose metabolism in patients with AD, as reported by Gejl *et al*.^27^. Restoration of mechanisms important to the neurovascular unit by liraglutide and prevention of further neurovascular degeneration would then avert the potential exacerbation of glucose transporter deficiency in AD^28^, in agreement with the view that glucose transport is a potentially important therapeutic target of treatment in AD^6,25^.

The rationales of the present paper are, first, the test of the physiological impact of BBB glucose transport on AD progress and evolution and, second, the test of restoration of mechanisms important to the neurovascular unit by liraglutide and prevention of further neurovascular degeneration would then avert the potential exacerbation of glucose transporter deficiency in AD, in agreement with the view that glucose transport is a potentially important therapeutic target of treatment in AD.

On the basis of the results of GLP-1 analog treatment, and the effects on CMR_glc_^27^, we tested the hypothesis that liraglutide is a GLP-1 analog that raises the *T*_max_ and the clearance from the circulation of glucose by the brain tissue. We determined the values of *T*_max_ and CMR_glc_ during AD evolution and progression, as reflected in measures of cognition and disease duration. Specifically, we tested the hypothesis that treatment with the GLP-1 analog liraglutide would prevent or reduce the decline of *T*_max_, assessed by PET of tracer [^18^F]FDG derived radioactivity in brain.

## Results

### Baseline

**Figure 1:**
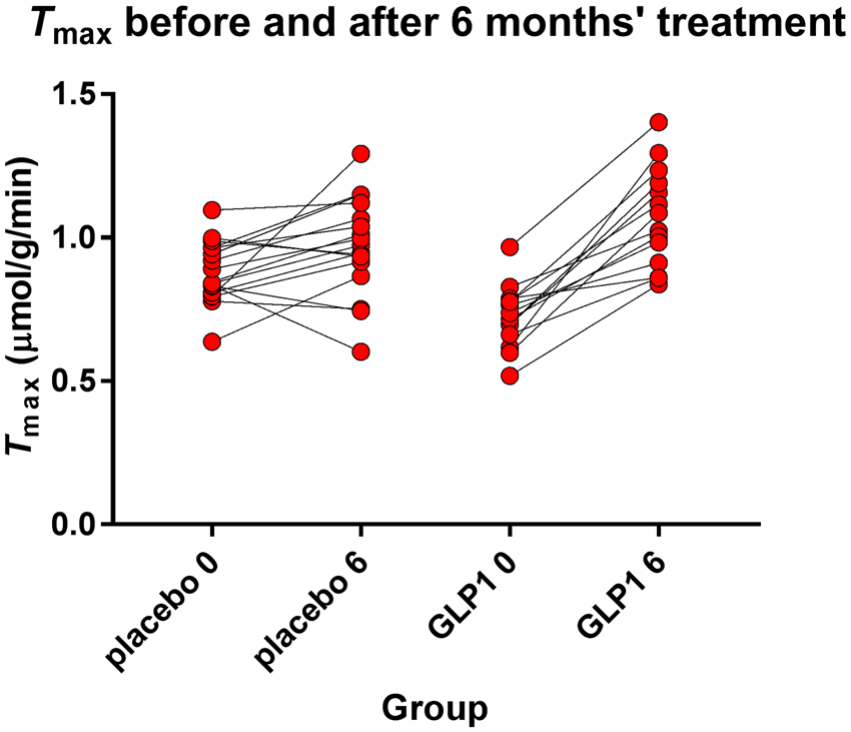
Estimates of *T*_max_ of two groups before and after liraglutide and placebo treatment. Ordinate *T*_max_ estimates. GLP-1 analog treatment very significantly (P < 0.0001) raised the average *T*_max_ estimate in cerebral cortex as a whole. The resulting value of *T*_max_ significantly exceeded the value reached by placebo treatment.

Group differences at baseline are listed by Gejl *et al*.^27^. Disease duration was significantly longer in the liraglutide group (30 ± 6 vs 15 ± 3 months). At baseline the average *T*_max_ estimate was significantly lower in the treatment group (P = 0.02), compared to the placebo group, with a mean difference of 0.15 µmol/g/min, 95% CI of diff: 0.29; 0.017), as shown in Fig. 1. The liraglutide group was slightly younger (63.1 vs. 66.6 years, P = 0.16), as was the group of six cognitively normal volunteers at 63 ± 3 years^29^.

### Group Regressions and Correlations at Baseline

**Figure 2:**
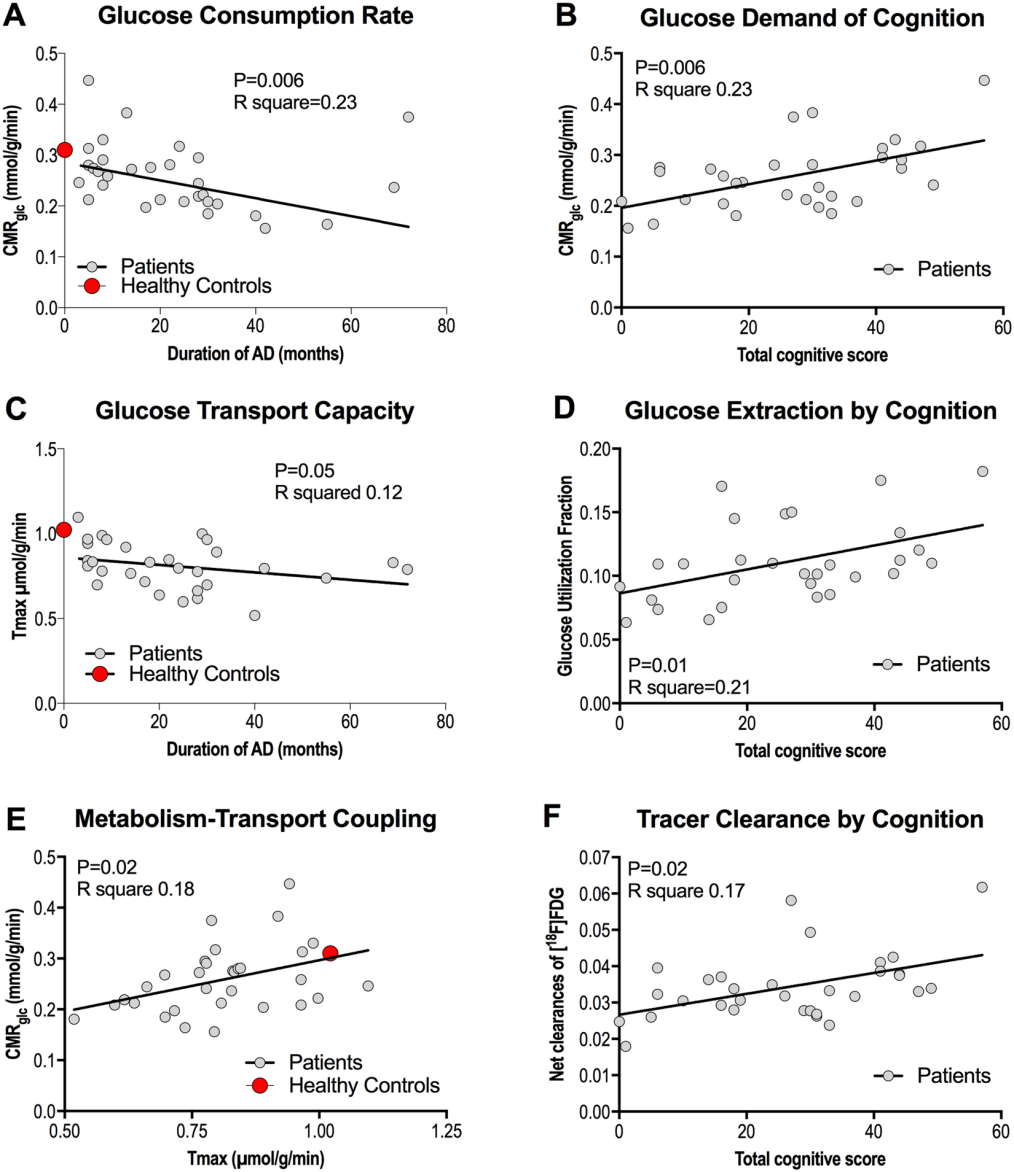
Relationship between duration of AD and CMR_glc_ (**A**), total cognitive score and CMR_glc_ (**B**), duration of AD and T_max_, (**C**), total cognitive score and GUF (**D**), T_max_ and CMR_glc_ (**E**), and total cognitive score and net clearance of [^18^F]FDG. The points representing the averages of the healthy control group were not included in the analysis.

Linear regressions among the variables determined at baseline are shown in Fig. 2: Analysis of CMR_glc_ versus duration of AD revealed a negative correlation (P = 0.006, R^2^ = 0.23, Fig. 2A) at baseline of both groups pooled. We observed a positive correlation between CMR_glc_ and the total cognition score at baseline of all members of the two groups pooled (P = 0.006, R^2^ = 0.23, Fig. 2B). We found tendencies of correlation between the *T*_max_ estimates and the duration of disease (P = 0.05, R^2^ = 0.12, Fig. 2C) at baseline of all members of the groups pooled, nor between the *T*_max_ estimates and total cognitive score (P = 0.83, R^2^ = 0.002, not shown). We found positive linear correlation of glucose utilization fraction (GUF) and cognition (P = 0.01, R^2^ = 0.21, Fig. 2D) at baseline of both groups pooled, but no correlation between GUF and duration of disease (P = 0.19, R^2^ = 0.06, not shown), also of all members of the two groups pooled, nor between the unidirectional glucose extraction fraction (GEF) and cognition or duration (P > 0.35, R^2^ < 0.03, not shown). We also found a positive linear correlation of CMR_glc_ and *T*_max_ (P = 0.02, R^2^ = 0.18, Fig. 2E).

We observed a positive correlation between the net clearance of [^18^F]FDG (and therefore of glucose as well) and the total cognitive score at baseline of both groups pooled (P = 0.02, R^2^ = 0.17, Fig. 2F). The correlation of the net clearance of [^18^F]FDG (and therefore of glucose) and the duration of AD was significantly negative (P = 0.007, R^2^ = 0.22, not shown) at baseline for the members of both groups pooled.

### Group Variables, Changes, and Differences During and After Treatment

Gejl *et al*.^27^ reported a decrease in fasting plasma glucose in both groups and a significant difference of fasting plasma glucose levels between the two groups after six months of treatment (5.6 mM in the placebo group and 5.1 mM in the GLP-1 analog group). Fasting plasma glucose levels in the heathy group was 5.8^30^. We also noted significant reduction of systolic and diastolic blood pressures in the liraglutide group at the end of the study period.

**Figure 3:**
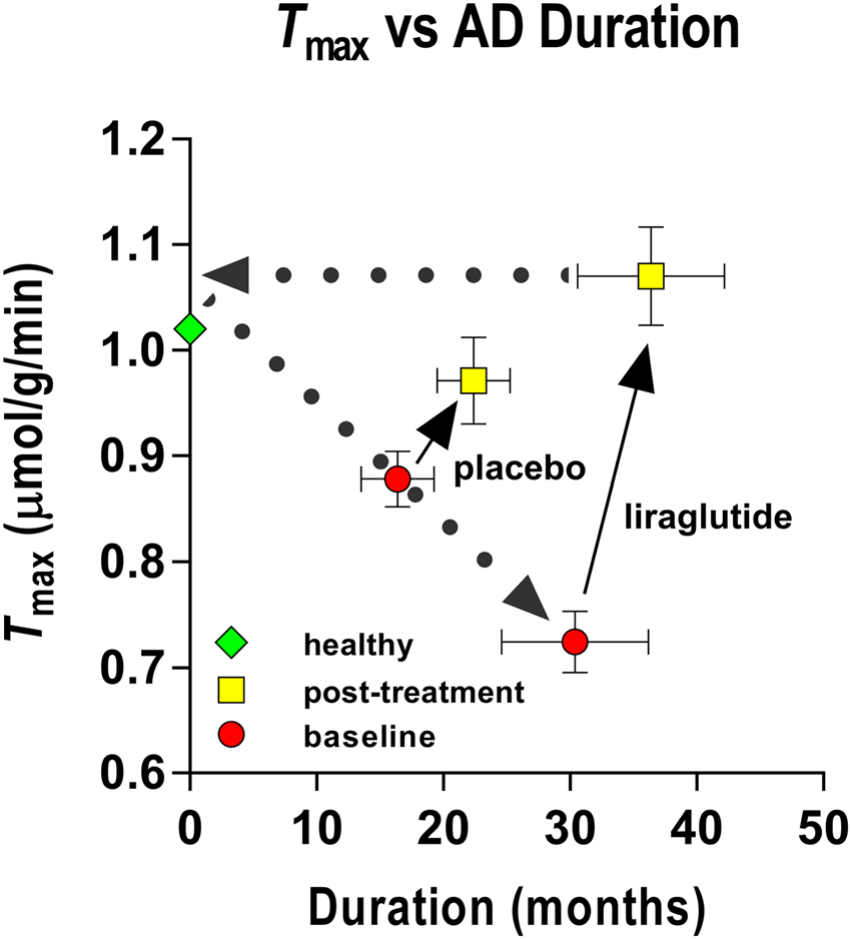
The GLP-1 analog treatment appeared to reduce the effects of disease duration. The results are consistent with the claims (1) that the maximum blood-brain transfer capacity declines with duration of Alzheimer’s disease, and (2) that GLP-1 analog treatment raises the GLUT1 activity in the barrier as a potential future target for treatment of the neurovascular dysfunction in Alzheimer’s disease. Estimates of *K*_*t*_ averaged 9.3 mM (± 0.28 RSDR, robust standard deviation of residuals) in the placebo treated group at baseline, 10.1 mM (± 0.19 RSDR) at six months, and 5.9 mM (± 0.18 RSDR) in the liraglutide group at baseline, and 11.1 mM (± 0.25 RSDR) at six months of treatment. The points representing the averages of the healthy control group were not included in the analysis.

We observed no change of the cortical *T*_max_ estimate (P = 0.24, mean difference 0.093 µmol/g/min, 95% CI of difference: −0.037; 0.22) in the placebo group during the six months of treatment. In the GLP-1 analog treatment group, the *T*_max_ estimate increased significantly after the 6 months of treatment (P < 0.0001, mean difference 0.34 µmol/g/min, 95% CI of the difference 0.20; 0.49), as shown in Fig. 1 of both estimates. After the six months, the *T*_max_ estimates of the two groups no longer differed significantly (P = 0.24). The estimates of *T*_max_ increased significantly more in the liraglutide treated group than in the placebo group (P = 0.0002, mean difference 0.25 µmol/g/min, 95% CI of the difference 0.13; 0.37), as shown in Figs 1 and 3. In healthy volunteers, the *T*_max_ estimates averaged 1.022 umol/g/min, as shown in Fig. 3.

**Figure 4:**
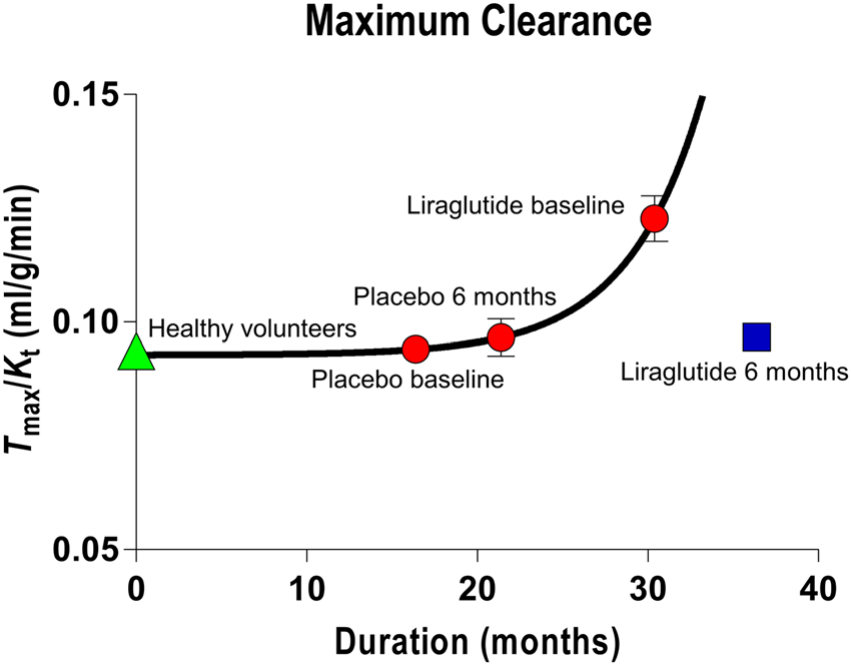
Maximum clearance as function of disease duration of all baseline and placebo treatment groups, and separately for liraglutide group treated for 6 months. Abscissa: Duration (months). Ordinate: Maximum clearance calculated as average of Tmax/Kt ratios for the four groups. Curve represents best fit of exponential growth equation Y = Y_o_ + A exp(−k(X − X_o_)) with R^2^ = 0.4 and parameters + standard errors of Y_o_ = 9.28 + 0.08 ml/hg/min, A = 0.12 + 0.49 ml/hg/min, and k = 0.23 + 0.27 min^−1^, with X_o_ fixed at 16.4 months, the average duration of the placebo group members at baseline. The points representing the averages of the healthy control group were not included in the analysis.

The maximum observable clearance is the ratio of an estimate of *T*_max_ to the corresponding estimate of the Michaelis half-saturation concentration *K*_*t*_, i.e., the ${T}_{{\rm{\max }}}/{K}_{t}$ ratio, equal to the abscissa intercept of the Eadie-Hofstee plot. The ratio is a measure of the highest attainable transport capacity at the present affinity of the transporters to glucose. The maximum clearance estimates increased with the duration of disease, as shown in Fig. 4 for the placebo group at baseline and 6 months, and for liraglutide treatment group at baseline. The six months of liraglutide treatment reversed the trend by returning the ratio to the value of the placebo group at baseline. The corresponding estimates of the Michaelis half-saturation concentration are listed in the legend to Fig. 1.

The estimates of net clearance of [^18^F]FDG and glucose (*K* ^*^ and *K*) did not change in the placebo group (P = 0.53), but increased with borderline significance in the liraglutide group (P = 0.049), as listed in Table 1. We calculated the estimates of CMR_glc_ listed in Table 1 by regional kinetic analysis. The estimates confirmed the decrease in the placebo group (P = 0.05) and unaltered CMR_glc_ in the liraglutide group (P = 0.58) reported by Gejl *et al*.^27^. The CMR_glc_ estimates of the healthy control group was 0.31 ± 0.062 umol/g/min^29^.

**Table 1:** Kinetic analysis results.

**Placebo**	Vb	K1*	K2*	K3*	Jglc	K	CMRglc	Ctissue	K2	J2	K1	GUF	GEF
mean baseline	0.066	0.094	0.107	0.062	0.375	0.035	0.268	1.448	0.073	0.107	0.064	0.115	0.165
sd	0.020	0.016	0.014	0.015	0.058	0.010	0.071	0.589	0.010	0.051	0.011	0.031	0.042
mean 6 month	0.062	0.097	0.117	0.062	0.370	0.033	0.248	1.547	0.079	0.122	0.065	0.106	0.160
sd	0.014	0.013	0.029	0.024	0.059	0.008	0.062	0.516	0.019	0.049	0.009	0.029	0.043
ttest	0.35	0.53	0.20	*0.37*	0.69	0.53	0.05	0.36	0.20	0.12	0.53	0.21	*0.49*
**Liraglutide**	**Vb**	K1*	K2*	K3*	**Jglc**	**K**	**CMRglc**	**Ctissue**	**K2**	**J2**	**K1**	**GUF**	**GEF**
mean baseline	0.065	0.102	0.131	0.064	0.383	0.034	0.246	1.531	0.089	0.137	0.069	0.106	0.165
sd	0.018	0.020	0.021	0.007	0.083	0.009	0.059	0.413	0.014	0.044	0.013	0.033	0.051
mean 6 month	0.069	0.105	0.117	0.066	0.361	0.038	0.250	1.382	0.079	0.111	0.071	0.126	0.179
sd	0.016	0.014	0.024	0.017	0.057	0.005	0.036	0.430	0.016	0.047	0.009	0.035	0.035
ttest	0.46	0.49	0.09	0.99	0.26	0.049	0.58	*0.36*	0.09	0.06	0.49	0.12	0.25

*paired test, nonparametric.

## Discussion

Analogs of the glucagon-like peptide-1 (GLP-1) hold promise as a novel approach to the treatment of neurodegenerative disorders such as AD^31^. The analogs target cerebral metabolism and neurodegeneration in animal models, with potential translation to afflicted humans^27^. In the present analysis of findings obtained in patients with AD, we discovered a highly significant effect of the GLP-1 analog liraglutide on the blood-brain glucose transport capacity. In the patients, the estimates of blood-brain glucose transport capacity correlated with the estimates of glucose consumption (CMR_glc_). We confirmed the natural disease progression in the study population, determined by a negative correlation between disease duration and neuronal activity measured as glucose metabolism and the tendency of the *T*_max_ estimates to decline with increasing duration of disease. The estimates of metabolism in turn correlated positively with measures of cognition, including the trend towards decreased estimates of cortical CMR_glc_ in the members of the placebo treatment group. The net clearances of [^18^F]FDG and glucose correlated positively with cognition and negatively with duration at baseline and increased significantly with treatment in the liraglutide group. The glucose utilization fraction (GUF) correlated positively with cognition and in the treatment group numerically exceeded the corresponding estimates of placebo group.

Glucose transport across the BBB is the net result of glucose fluxes in the two directions across both membranes of the capillary endothelium, mediated by glucose transporters of which GLUT1 predominates^32^. The fluxes across the membranes of the BBB can be regulated (1) by changing the concentration gradients in the directions of the tissue, thereby changing the differences between the fluxes in both directions across the endothelial membranes, (2) by changing the affinity of the transporters separately or jointly for the multiple substrates^33^, or (3) by changing the number or density of transporters by insertion of new proteins independently of the capillary surface area^34^. The last of the three mechanisms determines the magnitude of the maximum transport capacity (*T*_max_). The present results indicate that changes associated with the GLUT1 transporter can occur by changes of the density of GLUT1 or by changes of the half-saturation or affinity constants. In animal studies, reports suggest that regulation of GLUT1 is essential to preservation of proper brain capillary networking, blood flow, and endothelial integrity, as well as to neuronal function and structure^25^. Recent results imply that reduced glucose availability in the central nervous system directly triggers behavioral deficits related to the development of neuropathology and synaptic dysfunction mediated by hyperphosphorylated tau proteins^35^. The GLUT1 deficiency syndrome introduced by, among other authors of the report by DeVivo and Harik^36^, is an important example of an extreme version of GLUT1 deficiency that leads to a number of neurodegenrative features, of which Alzheimer’s disease may be said to be a milder example.

The present results agree with reports of healthy subjects^26^, although little is known of the specific effects of GLP-1 and its analogs on brain glucose transport. The claim of a direct effect of GLP-1 or its analogs on the transport of glucose across the BBB therefore remains speculative, but recent work by Jais *et al*.^37^ unveils the novel mechanism that vascular endothelial growth factor (VEGF) from macrophages at the BBB restores the presence of GLUT1 to baseline values, maintains CMR_glc_, and prevents loss of function. It is of particular interest that GLP-1 is coupled to augmented VEGF generation^38^. The most abundant GLUT in the BBB is GLUT-1, but low levels of other GLUTs (including the insulin sensitive GLUT-4) have been reported^6^.

The present observation of increased estimates of *T*_max_ in principle can be explained by increase of the number of GLUTs, prompted by liraglutide, or by subsequent increased postprandial insulin levels. Liraglutide raises GLUT-4 levels in the periphery via an AMPK-dependent mechanism that is independent of insulin^39^, but recent reports suggest that astrocytic insulin receptors modulate GLUT1 expression, and consequently GLUT1 protein levels at the cell membrane^40^. Moreover, insulin signalling in hypothalamic astrocytes supposedly contributes to glucose sensing in the central nervous system and systemic glucose metabolism by regulation of glucose uptake across the BBB^41^, in agreement with the present results, as GLP-1 potentially co-regulates the expression of hypothalamic insulin, although the mechanism is debated^42^. We note that liraglutide does not appear to cross the BBB of cerebral cortex^43^, suggesting direct effects on the barrier, or peripheral effects of receptor stimulation, e.g., anti-inflammatory^44^, potentially interfering with the reported decline of CMR_glc_ in AD.

The average value of the *T*_max_ of 1.02 umol/g/min in healthy volunteers agreed with values from Choi *et al*.^45^ who fitted the standard Michaelis–Menten equation to the measured brain glucose concentrations. As a function of plasma glucose, the regression yielded values of Michaelis constant *K*_*t*_ of 11.8 ± 1.6 mmol/L and a *T*_max_/CMR_glc_ ratio of 4.7 ± 0.14. Similarly, De Graaf *et al*.^46^ reported values of the *T*_max_/CMR_glc_ ratio that averaged 3.2 ± 0.10 and 3.9 ± 0.15 for gray matter and white matter using the standard transport model, with *K*_*t*_ of 6.2 ± 0.85 and 7.3 ± 1.1 mmol/L for gray matter and white matter. For an average whole-brain glucose consumption rate of 0.25 umol/g/min, the corresponding *T*_max_ estimates average close to 1 umol/g/min with an average Michaelis constant of 8.4 mmol/l. Direct *T*_max_ estimates by Brooks *et al*.; Feinendegen *et al*., and Blomqvist *et al*.^47–49^ averaged 0.9 umol/g/min, with an average Michaelis constant of 3.7 mM. Measurements of blood-brain unidirectional cleance of glucose (K1)^48–53^, averaged 0.08 ml/g/min for K1 and 0.34 umol/g/min for the blood-brain glucose flux (J1 = K1 Ca). The canonical *T*_max_ estimate for glucose transport across the human blood-brain barrier was listed by Gjedde^54^ as 1 umol/g/min.

The prevention of decline of CMR_glc_ is associated with an effect of liraglutide on the *T*_max_ estimates. The effect may represent a possible reversal of the expected down-regulation of the brain glucose transporters that are required for glucose uptake and metabolism in the brain^19^. We note that the finding of reduced glucose transport capacity in AD presented here may also to some extent reflect lower neuronal activity and/or loss of neuronal cell mass, raising the issue of the unresolved identification of cause and effect. Although the present population of patients only revealed a tendency towards a direct correlation with disease duration, animal studies show that BBB breakdown occurs before the development of functional deficits^25^. The reversal of the duration-related increase of the estimates of maximum clearance (${T}_{{\rm{\max }}}/{K}_{t}$) is a further indication that a potentially important mechanism of disease advance with duration is the increase of affinity of the transporters to glucose that causes the maximum clearance to increase, as the maximum transport capacity declines. The treatment with liraglutide reverses both of these trends, jointly expressed as a normalization of the estimates of maximum clearance.

Aging and neurodegenerative disorders have been shown to be associated with increased lactate concentration^55^, and lactate has been observed to play an important role in the regulation of cerebral blood flow^56,57^, implying that higher lactate production is associated with higher blood flow rates. One prediction from this association is higher blood flow rates relative to glucose consumption in these states, including lowering of the glucose utilization or net extraction fraction (GUF) in aging and neurodegenerative disorders. In agreement with these findings, we report a positive correlation between cognitive scores and GUF estimates in the patients from both groups at baseline. In addition, we report that the GLP-1 analog treatment is associated with insignificantly higher GUF estimates and significantly increased net clearances of [^18^F]FDG and glucose in the treatment group, compared to the placebo group, with numerical tendencies towards an increase of GUF estimates in the treatment group and a decline of GUF estimates in the placebo group (although neither trend is significant).

As predicted, the estimates of CMR_glc_ declined with disease duration, indicating the natural progression of the disease in this group of patients, signifying the conclusion that GLP-1 receptor stimulation halts the progression of Alzheimer’s disease, in association with a very significant increase of the *T*_max_ estimates. The decline of CMR_glc_ estimates coincided with the decline of cognitive functioning, as previously reported^11^. As the restoration of brain glucose levels and metabolism is likely to positively influence AD pathology^35^, this is a potentially novel approach to prevention or termination of disease progression.

### Limitations

The AD duration measure determined as the time from definite diagnosis naturally has considerable uncertainty. The fact that the inclusion criteria were solely clinical, potentially limits the accuracy of the duration measure^58^. A further caveat is the small subject sample sizes. Regardless of blinding and randomization, minor differences in the baseline characteristics of the treated and placebo groups influence subsequent disease progression and hence affect the therapeutic impact. In larger studies, randomization would be more likely to balance the treated and untreated groups. Despite the extensive evidence of the relation between CMR_glc_ and cognition, also as reported in a study of this size, the interpretation of measures of cognition in the current paper is of course speculative. While the trial was conducted as a randomized, placebo-controlled, double-blinded intervention study, and the PET analysis was carried out by an author blinded to the group and subject definitions (KV), the findings are the results of post-hoc analysis and as such were not obtained in a blinded fashion.

## Conclusion

In the present study, we report the evidence that prevention of the decline of CMR_glc_ is associated with improvement of BBB glucose transport capacity, detected as differences of estimates of the maximum capacity of blood-brain transfer of glucose (*T*_max_), interpreted as a crucial element of disease progression and duration, responsible for possible limitations of nutrient delivery. The evidence extends the previous discovery of an effect of GLP-1 on maximum blood-brain glucose transfer capacity in healthy human volunteers, reported by Gejl *et al*.^26^. The change of *T*_max_ estimates occurred in relation to changes of the net clearance of glucose, and net glucose consumption (CMR_glc_), both as functions of disease duration and cognitive ability. Thus, the restoration of brain glucose availability and neuronal metabolism with GLP-1 or an analog potentially protects against cognitive impairment in Alzheimer’s disease.

## Methods

### Study design and participants

In the present investigation, we completed a 26-week, randomized, placebo-controlled, double-blinded intervention with liraglutide or placebo in patients with AD, recruited from dementia clinics in Central Denmark, with key clinical inclusion and exclusion criteria and administrative details listed by Gejl *et al*.^27^.

We assigned 38 patients to receive either the GLP-1 analog liraglutide (n = 18) or placebo (n = 20), as described by Gejl *et al*.^27^. Of these, 14 patients had PET with [^18^F]FDG before and after treatment, compared to 19 patients who received placebo, all of whom completed the cognitive examination. Tomography sessions for CMR_glc_ were incomplete in two patients, leaving 17 patients from the placebo group and 14 patients from the liraglutide group in the final analysis of [^18^F]FDG-derived radioactivity. Demographic and clinical characteristics are described in Gejl *et al*.^27^. The AD duration measure was determined as the time from definite diagnosis.

Patients willing to participate gave written informed consent. Safety data were monitored independently throughout the study period. The study was conducted according to the principles of the Helsinki Declaration. The Central Denmark Regional Committees on Biomedical Research Ethics, the Danish Data Protection Agency, and the Danish Medicines Agency approved the protocol^59^, with trial registration at ClinicalTrials.gov: NCT01469351, November 1, 2011. Participants attended a screening visit to assess eligibility followed by randomization to liraglutide or placebo for 26 weeks. Liraglutide was administered as 0.6 mg subcutaneously daily for one week; hereafter 1.2 mg daily for one week, before final increase to 1.8 mg daily. The placebo group members received saline in similar volumes.

### Positron Emission Tomography

The subjects underwent PET of [^18^F]FDG uptake and metabolism, as described in previous dynamic [^18^F]FDG studies of brain metabolism^26,60^.

### Co-registration

We acquired anatomical images for co-registration with the 3 T Magnetom Tim Trio system (Siemens Healthcare, Erlangen, Germany) with 3D T1-weighted high-resolution anatomic scan of magnetization-prepared rapid acquisition gradient echo (MPRAGE) sequence. We co-registered PET images with individual MR images to an MR template, and evaluated the quality of each co-registration by visual inspection in 3 planes. PET and MR-images were co-registered and entered in Talairach space, and anatomical volumes of interest were used to extract time-activity-curves (TACs) from the dynamic PET images for the [^18^F]FDG analyses of cortex as a whole.

### Cognitive Testing

We evaluated cognition by the “Brief cognitive examination” from the Wechsler Memory Scale (WMS-IV)^61^, the test examines examining orientation, time estimation, mental control, clock drawing, incidental recall, inhibition and verbal reproduction.

### Kinetic Analysis

The primary outcome was the maximum glucose transport capacity of the BBB (*T*_max_.) assessed with [^18^F]FDG. We also determined magnitudes of unidirectional glucose extraction fraction (GEF) from measures of [^18^F]FDG uptake and blood flow, the latter as reported by Gejl *et al*.^27^. Using a 3-compartment model, we obtained values of ${K}_{1}^{\ast }$. (unidirectional clearance where symbols marked with asterisk indicate tracer [^18^F]FDG), ${k}_{2}^{\ast }$. (efflux rate constant), ${k}_{3}^{\ast }$ (phosphorylation rate constant) and ${V}_{b}$ (volume of brain occupied by intravascular blood) for [^18^F]FDG. The absolute dephosphorylation flux and the absolute quantity of [^18^F]FDG were both considered negligible during the scanning period. In the use of [^18^F]FDG to trace glucose metabolism, the “lumped constant” (LC) is a necessary isotope correction factor that we set to the value of 0.76^27^. T ratio between unidirectional clearances of [^18^F]FDG and glucose (τ) was set at the value of 1.48 and the phosphorylation ratio (ϕ) at the value of 09^62^. T net clearance of [^18^F]FDG then is given by^63^,

1 $${K}^{\ast }=\frac{{K}_{1}^{\ast }{k}_{3}^{\ast }}{({k}_{2}^{\ast }+{k}_{3}^{\ast })}$$

where *K* ^*^ and ${K}_{1}^{\ast }$ are the net and unidirectional blood-brain clearances of [^18^F]FDG, and ${k}_{2}^{\ast }$, and ${k}_{3}^{\ast }$ are the corresponding rate constants of [^18^F]FDG-derived radioactivity exchanges between circulation and the [^18^F]FDG precursor pool mediated by GLUT1 and hexokinase. The unidirectional glucose flux from blood into brain was calculated from the unidirectional [^18^F]FDG clearance as,

2 $${J}_{1}={K}_{1}^{\ast }{C}_{a}/\tau $$

where *J*_1_ is the unidirectional flux of glucose (rather than [^18^F]FDG) from blood to brain, *C*_a_ is the arterial plasma glucose concentration, and *τ* is the affinity ratio of [^18^F]FDG to glucose for blood-brain transfer across the BBB. The net clearance of glucose by definition is

3 $$K={K}^{\ast }/\mathrm{LC}$$

where ${\rm{LC}}$ is the “lumped constant”, defined above as the ratio of the net clearances of [^18^F]FDG and glucose, such that the net cerebral metabolic rate for glucose is calculated from the net [^18^F]FDG clearance as,

4 $${{\rm{CMR}}}_{{\rm{glc}}}={K}^{\ast }{C}_{a}/\mathrm{LC}\,$$

such that the cerebral tissue glucose concentration ${C}_{{\rm{tissue}}}$ is

5 $${C}_{{\rm{tissue}}}=\tau ({J}_{1}-{{\rm{CMR}}}_{{\rm{glc}}})/{k}_{2}^{\ast }$$

and the flux of glucose from brain to blood, ${J}_{2}$, is

6 $${J}_{2}={J}_{1}-{{\rm{CMR}}}_{{\rm{glc}}}$$

Here, the unidirectional glucose extraction fraction (GEF) by definition is ${K}_{1}/F\,\,$where *F* is cerebral blood flow, while the glucose utilization fraction (GUF), also by definition, is ${{\rm{CMR}}}_{{\rm{glc}}}/(F{C}_{a})$. We determined the blood-brain unidirectional clearance, defined as,

7 $${K}_{1}={J}_{1}/{C}_{a}$$

from the values of flux and concentration, while the glucose clearance from brain tissue to the circulation (*K*_2_) likewise was determined from the brain-blood flux and brain tissue concentration of glucose, as,

8 $${K}_{2}={J}_{2}/{C}_{{\rm{tissue}}}$$

### Michaelis-Menten analysis

Together, the two sets of values of clearance and corresponding fluxes (*K*_1_, *J*_1_ and *K*_2_, *J*_2_) depend on the affinity and maximum transport capacity of glucose across the BBB, as implemented twice in the linearized Eadie-Hofstee Plot version of the Michaelis-Menten equation^64–66^, both for the blood-brain transfer direction,

9 $${J}_{1}={T}_{{\rm{\max }}}-{K}_{t}\,{K}_{1}$$

and for the brain-blood transfer direction,

10 $${J}_{2}={T}_{{\rm{\max }}}-{K}_{t}\,{K}_{2}$$

from which we calculated the parameters *T*_max_ and *K*_*t*_ by multilinear regression, yielding a shared estimate of *K*_*t*_ for all members of the group and individual estimates of *T*_max_ for each member.

### Control group

We obtained a control estimate of the maximum glucose transport across the blood-brain barrier from published measurements by Kuwabara *et al*.^29^ that we analyzed in the same manner as the estimates in the four groups of patients with Alzheimer’s disease, after correction for the lower resolution and greater partial volume effect of PET before the 21st Century (radioactivities increased by 1.33 for cerebral cortex).

### Statistics

We analyzed treatment group data in two ways: We analyzed changes of kinetic variables by paired t-tests within groups, and changes of *T*_max_ with 2-way ANOVA of qui-squared with Tukey’s correction for multiple comparisons within and between groups. P-values less than 0.05 were considered indicative of significant difference. Spearman and Pearson’s r tests were used to evaluate correlations, implemented in GraphPad Prism (GraphPad Software, San Diego, CA) and PMOD (PMOD Technologies Ltd., Zürich, Switzerland).
